# Common network effect-patterns after monoamine reuptake inhibition in dissociated hippocampus cultures

**DOI:** 10.1007/s00702-022-02477-6

**Published:** 2022-02-24

**Authors:** Julia Trepl, Marc Dahlmanns, Johannes Kornhuber, Teja Wolfgang Groemer, Jana Katharina Dahlmanns

**Affiliations:** 1 Department of Psychiatry and Psychotherapy, Friedrich-Alexander University of Erlangen-Nürnberg, Schwabachanlage 6, 91054 Erlangen, Germany; 2 Institute for Physiology and Pathophysiology, Friedrich-Alexander University Erlangen-Nürnberg, 91054 Erlangen, Germany

**Keywords:** Hippocampal networks, Antidepressants, Hippocampus culture, Monoamine reuptake inhibitors, Connectivity

## Abstract

The pharmacological treatment of major depressive disorder with currently available antidepressant drugs is still unsatisfying as response to medication is delayed and in some patients even non-existent. To understand complex psychiatric diseases such as major depressive disorder and their treatment, research focus is shifting from investigating single neurons towards a view of the entire functional and effective neuronal network, because alterations on single synapses through antidepressant drugs may translate to alterations in the entire network. Here, we examined the effects of monoamine reuptake inhibitors on in vitro hippocampal network dynamics using calcium fluorescence imaging and analyzing the data with means of graph theoretical parameters. Hypothesizing that monoamine reuptake inhibitors operate through changes of effective connectivity on micro-scale neuronal networks, we measured the effects of the selective monoamine reuptake inhibitors GBR-12783, Sertraline, Venlafaxine, and Amitriptyline on neuronal networks. We identified a common pattern of effects of the different tested monoamine reuptake inhibitors. After treatment with GBR-12783, Sertraline, and Venlafaxine, the connectivity degree, measuring the number of existing connections in the network, was significantly decreased. All tested substances led to networks with more submodules and a reduced global efficiency. No monoamine reuptake inhibitor did affect network-wide firing rate, the characteristic path length, or the network strength. In our study, we found that monoamine reuptake inhibition in neuronal networks in vitro results in a sharpening of the network structure. These alterations could be the basis for the reorganization of a large-scale miswired network in major depressive disorder.

**Supplementary Information:**

The online version contains supplementary material available at 10.1007/s00702-022-02477-6.

## Introduction

Major depressive disorder (MDD) is a disease characterized by persistent feelings of sadness, guilt, and worthlessness (American Psychiatric Association 2013). With a worldwide lifetime prevalence averaging around 16.6% and a rising number of patients, the WHO projected MDD to become one of the most burdensome diseases in the western world (American Psychiatric Association 2017; GBD 2016 DALYs and HALE Collaborators 2017; Olesen et al. 2012). MDD also accounts for a great portion of the total health-related expenses with an annual cost of 100 billion Euro in Europe alone, implying not only an economic burden for society, but a threat to our social and health care systems (Olesen et al. 2012; Sobocki et al. 2006). Many currently available antidepressant drugs (ADDs) are selective monoamine reuptake inhibitors, which often take weeks to months to show clinical effects (Nestler et al. 2002; Katz et al. 2003; Rush et al. 2006). Given the rapid brain accumulation of drugs like fluoxetine, the underlying cause for the delayed treatment response is only insufficiently understood (Nestler et al. 2002).

For decades, the molecular research on neurological and psychiatric diseases was based on the single neuron as the structural and functional unit of the nervous system (Yuste 2015). While these investigations led to groundbreaking discoveries, neurons rarely act on their own—they rather function in networks through their interactions (Bassett and Sporns 2017). A single neuron receives various inputs from thousands of other neurons and can target an equally large number of neurons (Laughlin and Sejnowski 2003). Therefore, a change on only a few synapses can subsequently lead to changes in the whole neuronal network (Bassett and Sporns 2017; Yuste 2015). With the brain consisting of networks of different brain areas, groups of neurons, and individual neurons, it can be classified as a multiscale network system. Modifications of the composition of these networks at the molecular scale can have an impact on higher brain functions (Bassett and Sporns 2017). To this day, there is no general theory about the function of neuronal circuits and how their dysfunction might be the cause of mental or neurological diseases (Nestler et al. 2002; Yuste 2015). However, those diseases might derive from disturbances of neuronal networks on multiple scales with antidepressant drugs potentially reversing these disturbances (Bassett and Sporns 2017; Catani and ffytche 2005).

Networks of neurons are studied at many different levels and are presented in the mathematical form of a graph which consists of nodes (building blocks of the network—on different scales these may be single neurons, groups of neurons, or distinct brain areas) and edges (the connections linking the nodes) (Bassett and Sporns 2017; Wrosch et al. 2017). Depending on the nature of relationship between the network nodes that we focus on, we can generally distinguish three types of neuronal networks: Anatomical networks represent physical connections (Bullmore and Sporns 2009), functional networks describe the statistical dependence between the activities of two nodes without specifying the cause of correlation (Bullmore and Sporns 2009; Feldt et al. 2011), and finally, effective networks determine the influence that the activity of one node has on another node (Feldt et al. 2011; Bullmore and Sporns 2009). Functional and effective networks are based on statistical and information processing models. While of course also statistical relationships have to be rooted in physical connections on some level, these networks, however, do not require a direct anatomical connection from one cell to another for a functional or effective connection to occur.

Universal patterns of network structure, such as ‘small-worlds’ (Feldt et al. 2011; Bullmore and Sporns 2009; Latora and Marchiori 2001), can be affected in neurological and psychiatric diseases (Iturria-Medina et al. 2008; Achard and Bullmore 2007; Salvador et al. 2005; Guo et al. 2014). Neuroimaging studies showed specifically that major depressive disorder (Zhang et al. 2011; Cooney et al. 2010; Kaiser et al. 2015) and other mental disorders such as Alzheimer’s disease (Goveas Joseph et al. 2011) and schizophrenia (Jafri et al. 2008; van den Heuvel et al. 2013) affect neuronal network function on a whole-brain scale.

Antidepressant drugs like selective serotonin reuptake inhibitors (SSRI), selective serotonin-norepinephrine reuptake inhibitors (SSNRI), tricyclic antidepressants (TCA), and monoamine oxidase inhibitors (MAOI) target monoaminergic systems by enhancing serotonergic, noradrenergic, and partly dopaminergic inputs, leading to modulations in synaptic strength (Citri and Malenka 2008). Through intracellular signaling cascades, antidepressant drugs further might increase the conductance of post-synaptic receptors leading to more permanent strengthening of synapses (Citri and Malenka 2008; Andrade and Rao 2010). In parallel, structural changes might be induced through de novo synthesis of synaptic proteins which lead to enlargement of synapses, dendritic outgrowth, and branching and even the growth of new synapses (Citri and Malenka 2008; Pittenger and Duman 2008; Seo et al. 2014). In a similar way, weakening and reduction of synapses are possible (Citri and Malenka 2008).

As such antidepressant treatment can reverse pathological alterations of larger scale networks (Gudayol-Ferré et al. 2015), we hypothesize that antidepressant drugs and their molecular and cellular effects also change effective connectivity on micro-scale neuronal networks.

We found that, while different monoamine reuptake inhibitors have some individual effects on the networks, a common pattern of effects results from monoamine reuptake inhibition.

## Materials and methods

### Cell culture

Hippocampal neuronal cultures were prepared as previously described (Tischbirek et al. 2012; Welzel et al. 2010). Briefly, 1–3-day-old Wistar rats of any sex were sacrificed by decapitation in accordance with the guidelines of the State of Bavaria and with approval of the ethics committee of the FAU Erlangen-Nürnberg. Whole hippocampi where removed and transferred to ice-cold Hank’s salt solution. Afterwards, cells were washed, digested, dissociated, and centrifuged. Cells were plated onto Matrigel-coated glass coverslips, kept in growth medium, and incubated at 37 °C and 95% humidity until the experiments.

### Pharmacological treatments

After 8–10 days in culture, the growth medium was supplemented with GBR-12783 (10 µM), Sertraline (1 µM), Venlafaxine (50 µM), Amitriptyline (10 µM), or solvent for 48 h. These concentrations were based on the previous studies showing enhanced expression of synaptic protein levels and dendritic outgrowth at similar dosages (de Leeuw et al. 2020; Seo et al. 2014; Kajitani et al. 2012). The cultures were imaged subsequently. All substances were purchased from Tocris Bioscience (Wiesbaden-Nordenstadt, Germany).

### Live cell recording of neuronal networks

The cells were stained with the calcium-sensitive fluorescent dye Fluo-8-AM for 30 min while being kept at 37 °C. Afterwards, cells were washed and placed into the recording chamber, and filled with imaging buffer (in mM: NaCl 144, KCl 2.5, Glucose 10, HEPES 10, CaCl_2_ 2.5, MgCl_2_ 2.5). Recordings were made at room temperature on a Nikon TI-Eclipse inverted fluorescence microscope equipped with a tenfold, 0.45 NA objective (Nikon Instruments Europe, Düsseldorf, Germany) and a water-cooled EM-CCD camera (iXon Ultra 897, Andor, Belfast, Northern Ireland). Each recording of spontaneous activity (18.5 min) was concluded with an electric field stimulation with alternating polarity, and delivered through two parallel platinum electrodes. Images were recorded with Andor Solis software with exposure time of 1 ms and recording frame rate of 27.33 images/s, generating 31,156 frames per recording. Recordings were exported for analysis into tagged image file format containing 512 × 512 pixels of 16-bit monochromatic intensity values.

### Image processing and network reconstruction

**Fig. 1:**
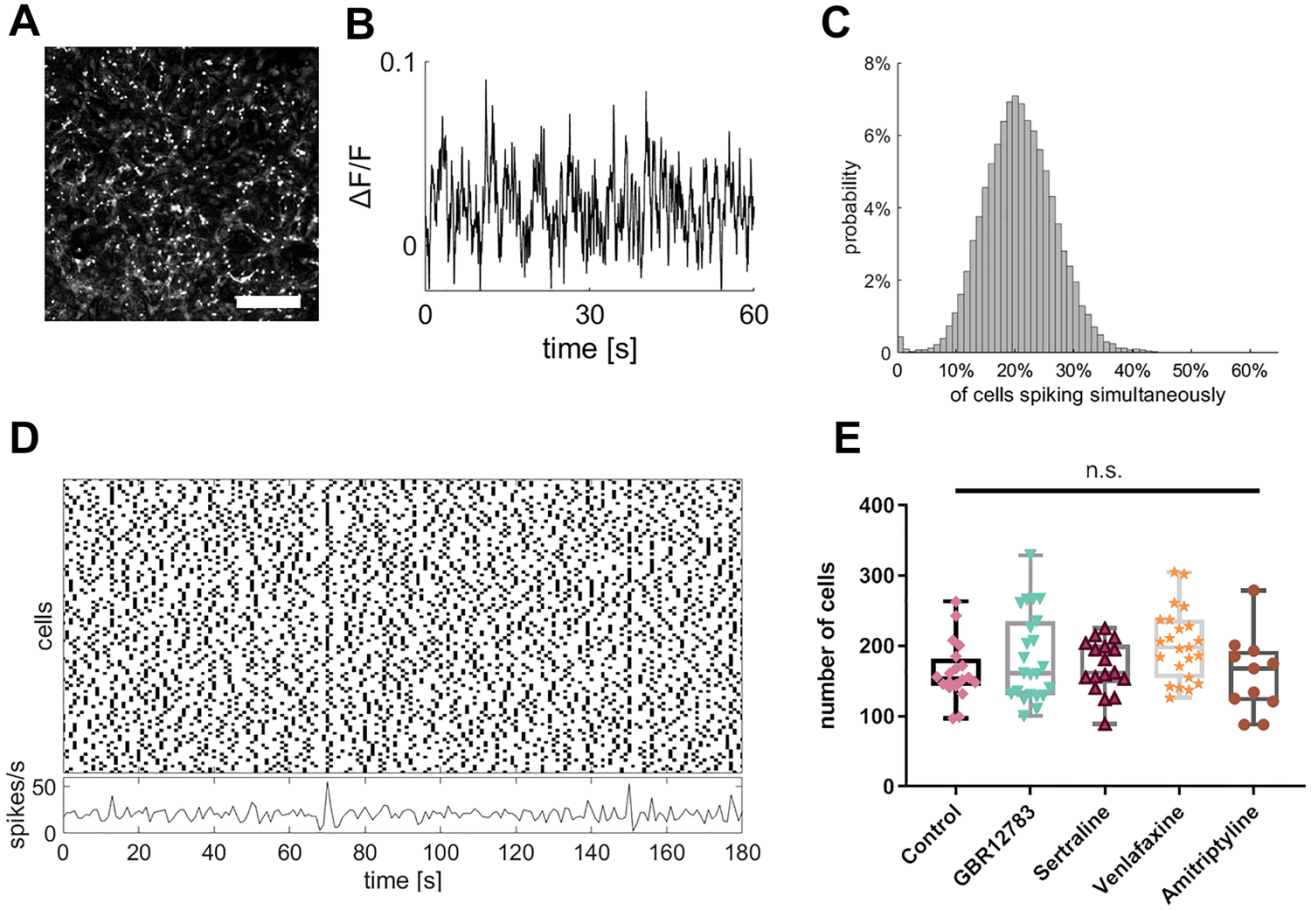
Excerpts of recording and processing steps from an exemplary culture. **a** Field of view taken from an exemplary culture (control 1). Scale bare = 500 µm. **b** Fluorescence trace of one single cell of the same culture registered over 1 min. **c** Participation in bursting behavior of all cells of control 1. **d** Spike traces of all cells of control 1 over 3 min. **e** There were no significant differences between groups regarding the mean number of cells. Monoamine reuptake inhibitors were present during incubation for 48 h. Sample sizes: control (*n* = 20), GBR12783 (10 µM, *n* = 23); Sertraline (1 µM, *n* = 17); Venlafaxine (50 µM, *n* = 23); Amitriptyline (10 µM, *n* = 11). The boxes extend from the 25th to the 75th percentiles. The median is shown as the horizontal line. The whiskers show the range of values

We used the fluorescence increase after electrical stimulation at the end of the recordings to filter for excitable neurons. Neuronal cell was detected using a feature point detection algorithm (Sbalzarini and Koumoutsakos 2005) and the fluorescence signals during the spontaneously captured recording phase were extracted from the image stacks. The fields of view in all recordings contained around 100–300 cells (Fig. 1E). The relative fluorescence traces were calculated for each cell (Jia et al. 2010).

Action potentials were estimated from the relative fluorescence traces with a template fitting and peeling algorithm (Deneux et al. 2016). This spike estimation yielded a binary time log for each cell with zeros (indicating no spiking activity) and ones (indicating spikes) in the different time frames. Based on these binary spiking data, we reconstructed effective neuronal networks in the cultures with a previously published machine learning model (Wrosch et al. 2017). A number of methods have been proposed to infer connectivity from spiking activity (Salinas and Sejnowski 2001; Xu et al. 1997; Stetter et al. 2012; Wrosch et al. 2017; Lungarella et al. 2007; Schreiber 2000). This approach uses eight of the best performing algorithms and combines them to a joint measure, which (on the simulated test data set) outperformed each individual algorithms prediction accuracy and sensitivity. This model is based on excitatory activity in the networks and validated with extensive simulations of varying connectivity (ranging from 0.05 to 0.8) and activity patterns (sparse to no bursting with an average spike propagation probability across a connection of 0.05 up to heavy bursting behavior with an average propagation probability of 0.8). The processing yields a binary, directed network (meaning the model predicts whether a specific connection from cell A to cell B exists, or not). For all predicted connections, the connection weight is calculated as the probability of spike propagation across this connection in the respective recording. This finally results in weighted, directed networks that we here analyzed for their structural and functional network properties.

### Graph theoretical network analysis

To analyze spiking properties of treated networks, the firing rate was calculated as number of spikes per second. To assess activity synchrony Cohen’s kappa, comparing the observed proportion of coincident spikes to the expected proportion of coincident spikes, was used (Illes et al. 2009; Cohen 1960). Graph theoretical network parameters were calculated using the brain connectivity toolbox (Rubinov and Sporns 2010). The most basic network property is the connectivity degree, the percentage of existing connections out of all possible connections. The strength of a connection in the network was defined as the probability of propagation of a spike in the source cell to a spike in the target cell within the next time frame (36 ms) and the average connection strength across each network (‘network strength’) was calculated.

Parameters defining the structure of the network can be divided into measures of functional integration and of functional segregation. Measures of functional integration give information about the communication between groups of neurons and their ability to integrate information (Rubinov and Sporns 2010). They are based on the shortest path length, which is defined as the minimal numbers of links connecting two nodes in the network (Feldt et al. 2011). Regarding the whole network, the characteristic path length is the average shortest path between two random nodes in a network (Feldt et al. 2011). It can be calculated with binary links or links that are weighted according to their connection strength (Wrosch et al. 2017). If every cell were directly connected with every other cell, the characteristic path length would be 1, implying maximum communication between specialized groups and therefore maximum integration of information (Rubinov and Sporns 2010). Inversely related to the average shortest path length is the global efficiency, giving information about the efficiency of parallel signal transfer across the network (Rubinov and Sporns 2010; Bullmore and Sporns 2009). If one node and its links are deleted, there is a drop in global efficiency, which is measured as the vulnerability to the loss of that node. The vulnerability of the entire network was defined as the mean efficiency loss across all nodes (Latora and Marchiori 2005; Newman 2003).

Measures of functional segregation, on the other hand, determine the existence of densely interconnected regions called clusters or modules which are able to process specialized information (Rubinov and Sporns 2010). The local clustering coefficient is calculated as the fraction of connections between the nearest neighbors of a node out of all possible connections (Feldt et al. 2011), meaning that if the connection partners of a certain cell are also highly connected with each other, the clustering coefficient is high. This clustering can be analyzed taking connection weights into account (weighted networks) or not (binary networks). The modularity degree reveals to which extent the network can be divided into modules of cells that are highly interconnected within members of the module, but have few connections with other modules (Rubinov and Sporns 2010). Complementing the structure analysis is the centrality of a node, defined as the number of shortest paths that pass through that node. This reflects the importance of the node in regard to information flow (Rubinov and Sporns 2010; Boccaletti et al. 2006; Nigam et al. 2016): Nodes with high centrality play a central part in integrating information between different subgroups of networks (Nigam et al. 2016; Schroter et al. 2017).

Network parameters were calculated using the following formulas:

Activity synchrony

$$K = \frac{1}{{N \times \left( {N - 1} \right)}} \times \mathop \sum \limits_{{\begin{array}{*{20}c} {i,j \in N} \\ {i \ne j} \\ \end{array} }} \frac{{\frac{{2 \times c_{ij} + N - s_{i} - s_{j} }}{N} - \frac{{s_{i} \times s_{j} + \left( {N - s_{i} } \right) \times \left( {N - s_{j} } \right)}}{{N^{2} }}}}{{1 - \frac{{s_{i} \times s_{j} + \left( {N - s_{i} } \right) \times \left( {N - s_{j} } \right)}}{{N^{2} }}}}.$$

With *N* denoting the number of cells in the network, *s*_*i*_ and *s*_*j*_ denoting the number of spikes in cells *i* and *j*, and *c*_*ij*_ denoting the number of synchronous spikes in cells *i* and *j*.

Connection strength

$$S_{ij} = \frac{{c_{ij} }}{{s_{i} }}.$$

Connectivity degree

$$c = \frac{{\mathop \sum \nolimits_{i,j \in N} a_{ij} }}{{N \times \left( {N - 1} \right)}}.$$

With *a*_*ij*_ denoting the connection between cells *i* and *j*.

Binary characteristic path length

$$L = \frac{1}{N} \times \mathop \sum \limits_{i \in N} \frac{{\mathop \sum \nolimits_{j \in N,j \ne i} d_{ij} }}{N - 1}.$$

With *d*_*ij*_ denoting the distance between cells *i* and cells *j*.

Weighted characteristic path length

$$L^{w} = \frac{1}{N} \times \mathop \sum \limits_{i \in N} \frac{{\mathop \sum \nolimits_{j \in N,j \ne i} d_{ij}^{w} }}{N - 1}.$$

With *d*^*w*^_*N*ij_ denoting the weighted distance between cells *i* and cells *j*.

Global efficiency

$$E^{w} = \frac{1}{N} \times \mathop \sum \limits_{i \in N} \frac{{\mathop \sum \nolimits_{j \in N,j \ne i} \frac{1}{{d_{ij}^{w} }}}}{N - 1}.$$

Clustering coefficient

$$C = \frac{1}{N} \times \mathop \sum \limits_{i \in N} \frac{{\frac{1}{2} \times \mathop \sum \nolimits_{j,h \in N} \left( {a_{ij} + a_{ji} } \right)\left( {a_{ih} + a_{hi} } \right)\left( {a_{jh} + a_{hj} } \right)}}{{\left( {k_{i}^{out} + k_{i}^{in} } \right) \times \left( {k_{i}^{out} + k_{i}^{in} - 1} \right) - 2\mathop \sum \nolimits_{j \in N} a_{ij} \times a_{ji} }};$$

with *k*^in^ denoting the in-degree (number of inward-links connecting to the cell) and *k*^out^ denoting the out-degree (number of outward-links connecting to the cell).

Modularity degree

$$Q = \frac{1}{l} \times \mathop \sum \limits_{i,j \in N} \left( {a_{ij} - \frac{{k_{ij}^{out} \times k_{ij}^{in} }}{l}} \right) \times \delta \left( {m_{i} ,m_{j} } \right);$$

with *l* denoting the number of links in the network and *m*_*i*_ denoting the module affiliation according to a generalized Louvain community (Leicht and Newman 2008).

Betweenness centrality

$$b_{i} = \frac{1}{{\left( {N - 1} \right) \times \left( {N - 2} \right)}} \times \mathop \sum \limits_{{\begin{array}{*{20}c} {h,j \in N} \\ {h \ne i,h \ne j,j \ne i} \\ \end{array} }} \frac{{\rho_{hj} \left( i \right)}}{{\rho_{hj} }};$$

with *ρ*_*hj*_(*i*) denoting the number of shortest paths between cells h and j that pass through cell i.

Vulnerability

$$V\left( i \right) = \frac{{E^{w} - E^{w} \left( i \right)}}{{E^{w} }};$$

with *E*^*w*^ denoting the global efficiency and *E*^*w*^(*i*) denoting the global efficiency after removal of node i and all its edges.

### Random networks

Data from random networks were included in the data plots as a reference. 100 random networks were generated by creating an adjacency matrix of the average network size in this study (153 cells) and assigning 5814 randomly placed connections (self-connections on the diagonal were prohibited), yielding a connectivity degree of 0.25—the average across networks in this study. For each connection, the connection weight was set, using normally distributed random numbers with mean 0.41 and standard deviation 0.03 (values derived from the entire population of connection weights in this study).

### Statistical analysis

Data resulting for network analyses were statistically analyzed using GraphPad Prism 7.0 software and are here shown as mean ± standard error of the mean (SEM). To compare control and experimental groups, a one-way ANOVA test with post hoc Dunnett’s tests was used. A probability level of *p* < 0.05 (*), *p* < 0.01 (**), or *p* < 0.001 (***) was assumed as significant.

## Results

**Fig. 2:**
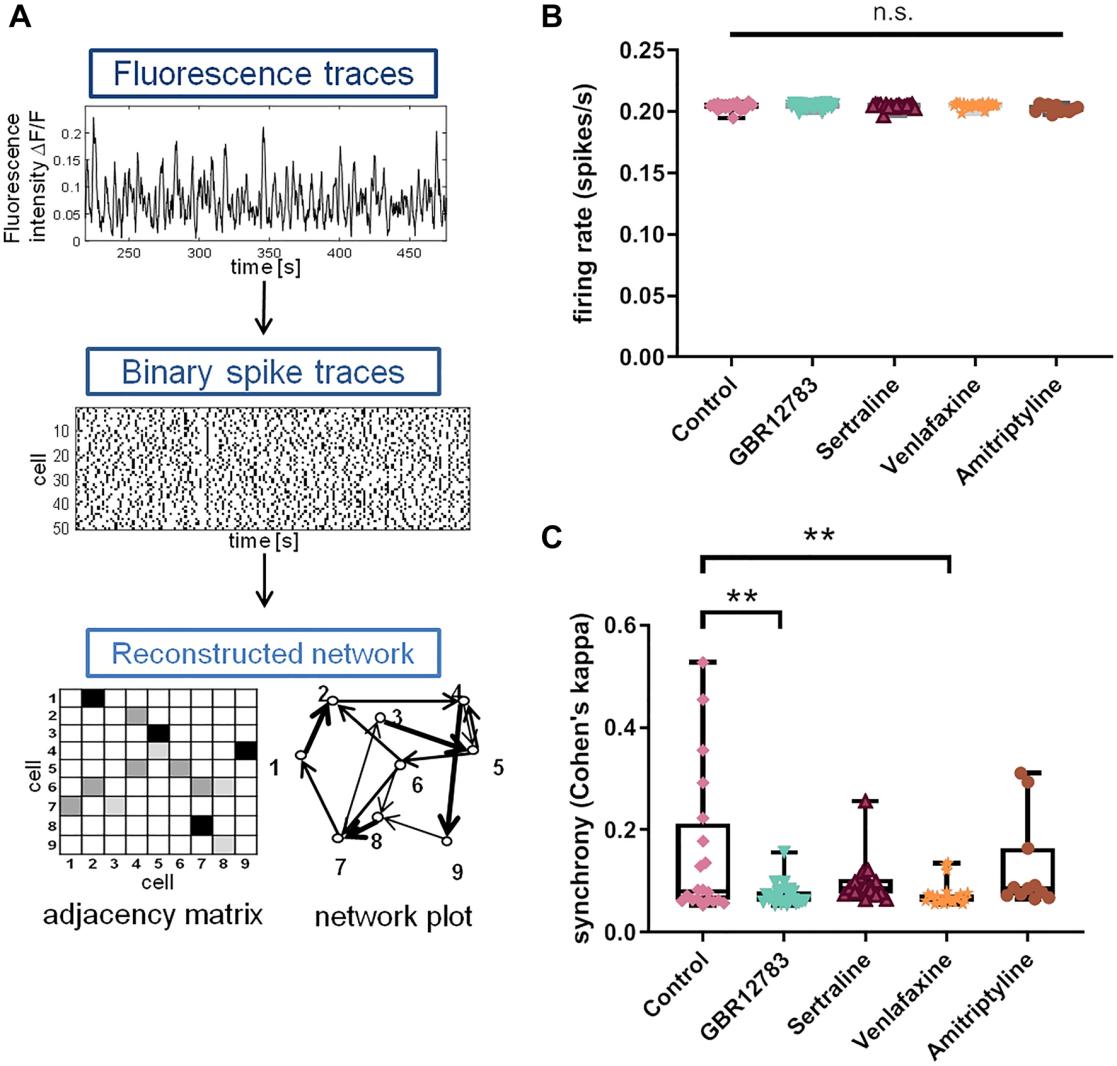
Reduced synchrony in neuronal networks following treatment with monoamine reuptake inhibition. **a** Schematic depiction of analysis pipeline consisting of recording of spontaneous activity with calcium imaging, detection of neuronal spikes with a template fitting algorithm, and reconstruction of neuronal networks with a machine learning model (Wrosch et al. 2017). Exemplary data are taken from a control culture. Fluorescence trace depicts a single cell, while rasterplot, adjacency matrix, and network illustration show an excerpt of 50 and 10 cells of the respective control culture network. **b** Overall firing rates of the cultures are maintained when treated with different monoamine reuptake inhibitors GBR12783, Venlafaxine, Sertraline, and Amitriptyline compared to control. **c** Synchrony of firing assessed as Cohen’s kappa (interrater variability) was significantly reduced compared to untreated control networks after treatment with monoamine reuptake inhibitors GBR12783 and Venlafaxine, but not after Sertraline and Amitriptyline. **p < 0.01 after one-way ANOVA followed by Dunnett’s post hoc test. Monoamine reuptake inhibitors were present during incubation for 48 h. Sample sizes: control (*n* = 20), GBR12783 (10 µM, *n* = 23); Sertraline (1 µM, *n* = 17); Venlafaxine (50 µM, *n* = 23); Amitriptyline (10 µM, *n* = 11). The boxes extend from the 25th to the 75th percentiles. The median is shown as the horizontal line. The whiskers show the range of values

To investigate how major depressive disorder therapy-relevant monoamine reuptake inhibition affects the structure and dynamics of neuronal networks, we pursued a previously published approach of extracting the cell-to-cell connectivity of in vitro neuronal network behavior from live-cell calcium fluorescence recordings in rat hippocampal cultures (Wrosch et al. 2017). Primary hippocampal cultures from newborn rats were prepared (Welzel et al. 2010; Tischbirek et al. 2012) and incubated until mature (8–10 days). After a subsequent 48 h treatment with the different test substances, fluorescence recordings with calcium-indicator dye Fluo-8 were recorded. An exemplary field of view and fluorescence trace are shown in Fig. 1A, B. These recordings were subjected to a cell detection and a spike estimation analysis and further processed by network reconstruction algorithms (Fig. 2A): In brief, we used the recorded spiking activities for each cell to reconstruct neuronal connectivity with a previously published machine learning model that combines different statistical algorithms to evaluate the cells’ spiking patterns for correlations and causal spike propagation relationships (Wrosch et al. 2017). This model is based on excitatory activity in the networks and validated with extensive simulations of varying connectivity and activity patterns. The processing yields a binary, directed network (meaning the model predicts whether a specific connection from cell A to cell B exists, or not). For all predicted connections, the connection weight is calculated as the probability of spike propagation across this connection, which finally results in weighted, directed networks that we here analyzed for their structural and functional network properties.

To verify that varying firing behavior of differentially treated networks does not confound further analysis, we first analyzed the spiking properties of cultures treated with different monoamine reuptake inhibitors: GBR12783, Sertraline, Venlafaxine, and Amitriptyline. We found that firing of the cells was around 0.2 spikes/s independent of pharmacological interventions (one-way ANOVA: F(4,89) = 2.136, *p* = 0.083; Fig. 2B). The participation in bursting events was similar across cultures of all conditions. Figure 1C shows an exemplary distribution of simultaneous spiking participation. The histogram shows a strong mode at very few cells spiking simultaneously with a slight tail to the right, which represents the sparse bursting events. This activity pattern of spontaneous activity with sparse spiking events is illustrated in a rasterplot in Fig. 1D.

### Individual effects of different monoamine reuptake inhibitors on neuronal network parameters

Synchronized firing during bursting events is an important feature of physiological hippocampal activity especially for information propagation within groups of neurons (Takahashi et al. 2010). We quantified the firing synchrony and found that both GBR12783 and Venlafaxine reduced spiking synchrony compared to untreated networks (one-way ANOVA: F(4,89) = 4.254, p = 0.0034, Dunnett’s post hoc test: *p* = 0.003 for GBR127832 and *p* = 0.003 for Venlafaxine; Fig. 2C). Amitriptyline, however, did not influence spiking synchrony (Dunnett’s post hoc test: *p* = 0.724), indicating, that—although equally effective in treating symptoms of major depressive disorder—not all monoamine reuptake inhibitors have the same effects on neuronal network behavior on a cellular level. Monoamine reuptake inhibitors suppress high-synchrony bursting behavior in thalamic neurons (Pape and McCormick 1989). Also here, in hippocampal cultures, the high synchrony expressed in some untreated networks (see Fig. 2C) does not occur under treatment conditions. To ensure that this effect does not influence the further investigated network parameters, we determined the correlation of network activity synchrony with some of the reconstructed network parameters. The synchrony is correlated with modularity with an R^2^ of only 0.07 (see supplementary figure S1). Connectivity degree (*R*^2^ = 0.16) and global efficiency (*R*^2^ = 0.28) are lightly correlated with the synchrony (see supplementary figures S2 and S3), which seems clear as an efficient and highly connected network is necessary for coordinated bursting (and high synchrony) to occur.

**Fig. 3:**
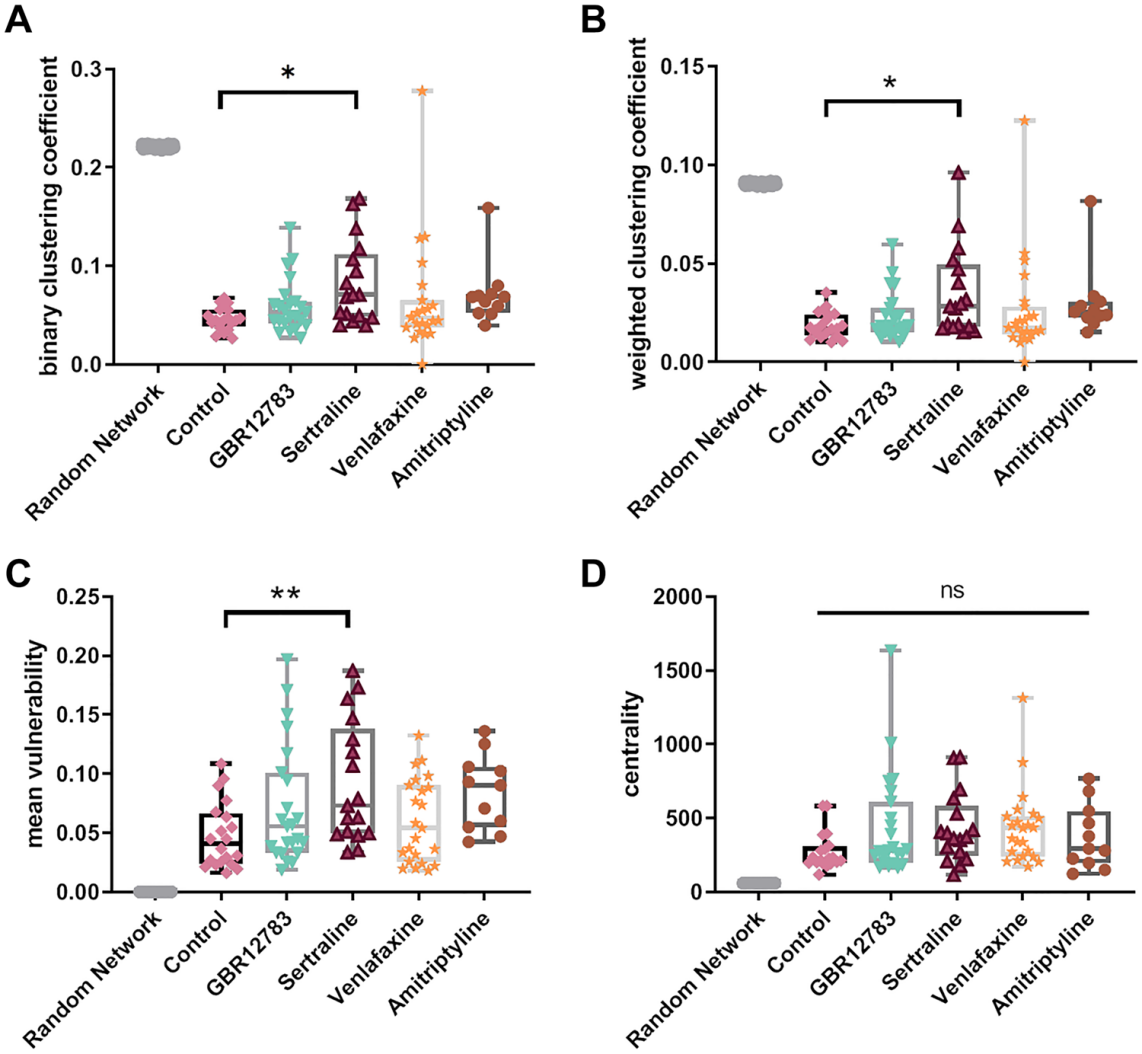
Individual Monoamine Reuptake Inhibitors have different effects on neuronal networks. **a-c** Specific network effects found after treatment with Sertraline, but not the other testes’ monoamine reuptake inhibitors: **a-b** Binary and connection strength-weighted clustering coefficients, quantifying strong local connectedness are enhanced after Sertraline treatment compared to controls, but not after treatment with the other monoamine reuptake inhibitors GBR12783, Venlafaxine, and Amitriptyline. **c** The average network vulnerability was calculated when computationally removing single neurons from the network and examining how well the network can compensate for such loss. Data show increased vulnerability only after treatment with Sertraline when compared to control, but not after other monoamine reuptake inhibitors. **d** Network centrality did not change after treatment with monoamine reuptake inhibitors. *p < 0.05, **p < 0.01 after one-way ANOVA followed by Dunnett’s post hoc test. Monoamine reuptake inhibitors were present during incubation for 48 h. Data from simulated random networks were added for comparison. Sample sizes: control (*n* = 20), GBR12783 (10 µM, *n* = 23); Sertraline (1 µM, *n* = 17); Venlafaxine (50 µM, *n* = 23); Amitriptyline (10 µM, *n* = 11). The boxes extend from the 25th to the 75th percentiles. The median is shown as the horizontal line. The whiskers show the range of values

In a similar fashion, we found, that of the tested compounds only the serotonin reuptake inhibitor Sertraline affected clustering coefficient as a measure of the local interconnectedness of the networks. Clustering based on the general existence of connections (binary clustering coefficient) (one-way ANOVA: F(4,89) = 2.33, *p* = 0.062; Dunnett’s post hoc test: *p* = 0.016; Fig. 3A), as well as the weighted clustering coefficient, where the different connections’ strength are integrated, is effectively enhanced by Sertraline (one-way ANOVA: F(4,89) = 2.19, *p* = 0.0763, Dunnett’s post hoc test: *p* = 0.024; les. 3B).

Similar to the increased clustering, the network vulnerability to a (computationally simulated) loss of single neurons is enhanced after treatment with Sertraline (one-way ANOVA: F(4,89) = 3.396, *p* = 0.0124; Dunnett’s post hoc test: *p* = 0.005 Fig. 3C). Treatment with other monoamine reuptake inhibitors did not induce strong changes regarding this parameter (Dunnett’s post hoc test *p* > 0.05 for all comparisons except with Sertraline). Thus, we identified that some properties of neuronal networks are modulated by individual classes of monoamine reuptake inhibitors.

### Common pattern of action of different monoamine reuptake inhibitors

**Fig. 4:**
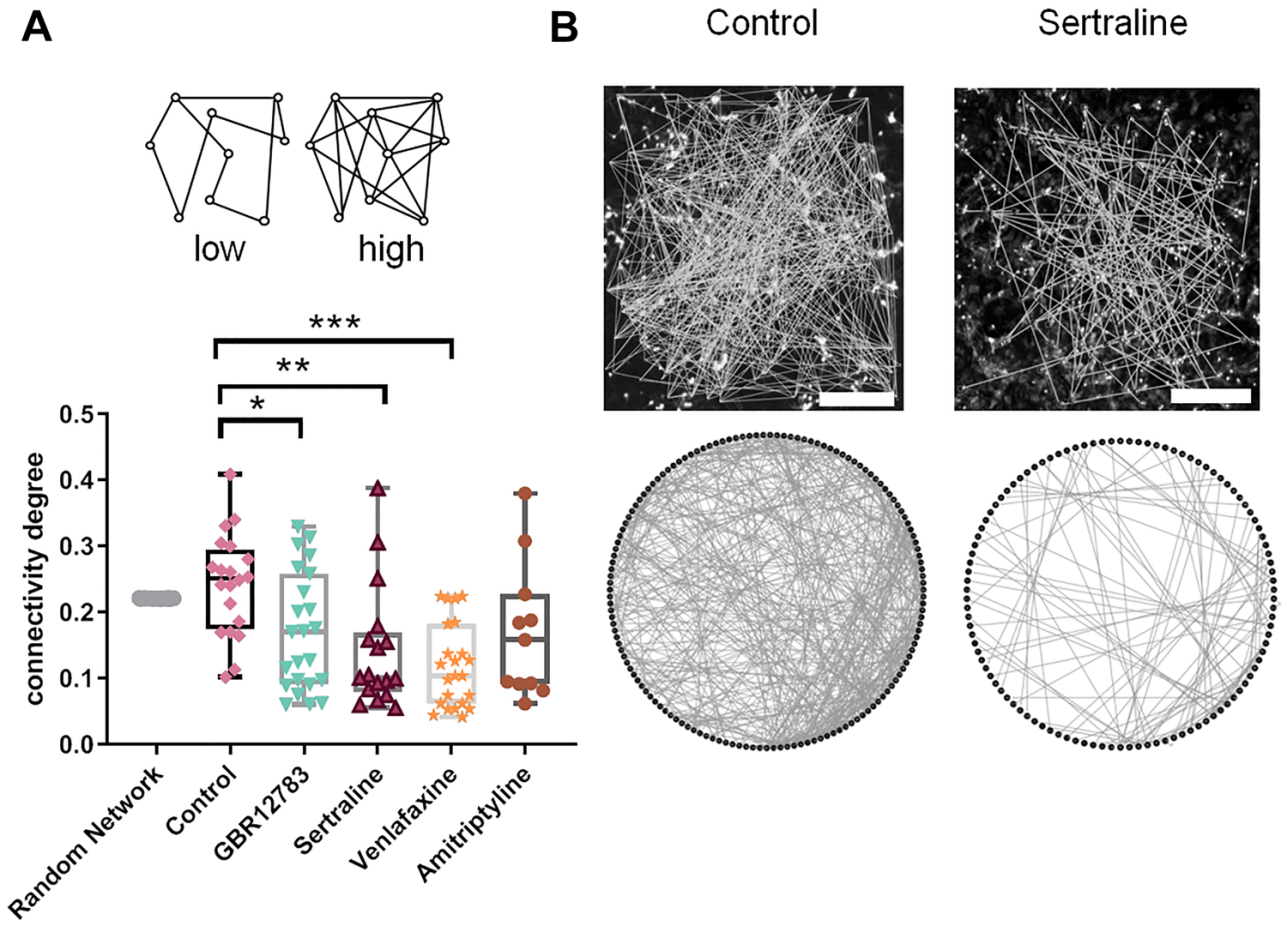
Monoamine Reuptake Inhibitors reduce network connectivity degree. **a** The connectivity degree quantifies the number of connections, normalized to the number of neurons in the network. Treatment with GBR12783, Sertraline, and Venlafaxine decreased the connectivity degree. **b** The upper panel shows two exemplary calcium fluorescence recordings (stained with Fluo-8) with similar number of cells in the field of view (control 143, Sertraline 143) and their reconstructed networks overlaid. After Sertraline treatment, the number of connections in the neuronal networks decreased compared to untreated control networks. The bottom panel illustrates the same networks in a degree-sorted circle layout. *p < 0.05, **p < 0.01, ***p < 0.001 after one-way ANOVA followed by Dunnett’s post hoc test. Monoamine reuptake inhibitors were present during incubation for 48 h. Data from simulated random networks were added for comparison. Sample sizes: control (*n* = 20), GBR12783 (10 µM, *n* = 23); Sertraline (1 µM, *n* = 17); Venlafaxine (50 µM, *n* = 23); Amitriptyline (10 µM, *n* = 11). The boxes extend from the 25th to the 75th percentiles. The median is shown as the horizontal line. The whiskers show the range of values

**Fig. 5:**
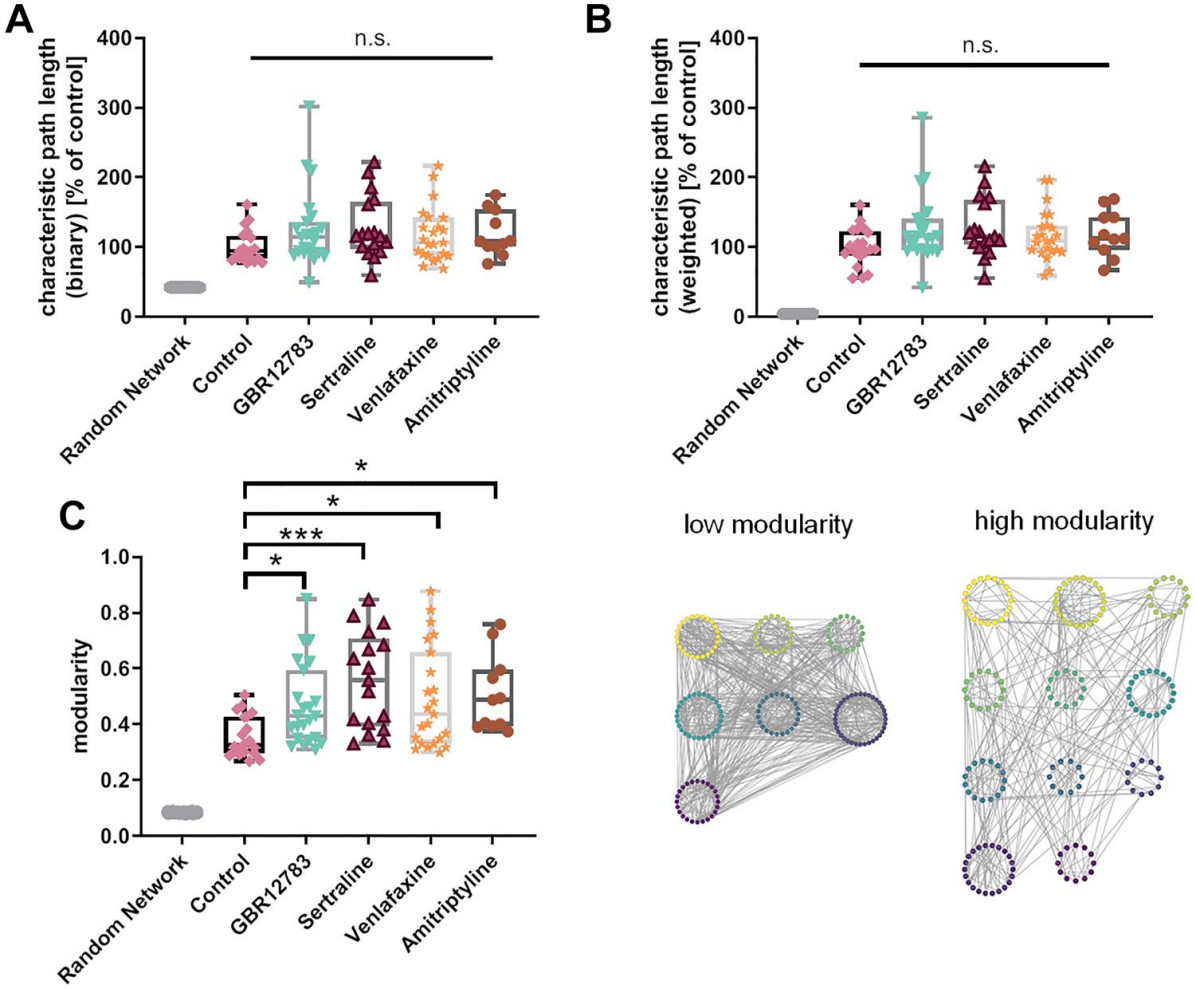
All tested Monoamine Reuptake Inhibitors increase networks’ modularity degrees. **a-b** Inhibition of monoamine reuptake does not lead to a change of the characteristic path length, neither of the binary, nor the weighted form (b). **c** All substances increase the modularity degree. Bottom shows to recorded networks with the same number of cells (*n* = 171) and low/high modularity (left control condition, right Sertraline condition). Cells are color-coded and grouped according to module affiliations. *p < 0.05, ***p < 0.001 after one-way ANOVA followed by Dunnett’s post hoc test. Monoamine reuptake inhibitors were present during incubation for 48 h. Data from simulated random networks were added for comparison. Sample sizes: control (*n* = 20), GBR12783 (10 µM, *n* = 23); Sertraline (1 µM, *n* = 17); Venlafaxine (50 µM, *n* = 23); Amitriptyline (10 µM, *n* = 11). The boxes extend from the 25th to the 75th percentiles. The median is shown as the horizontal line. The whiskers show the range of values

**Fig. 6:**
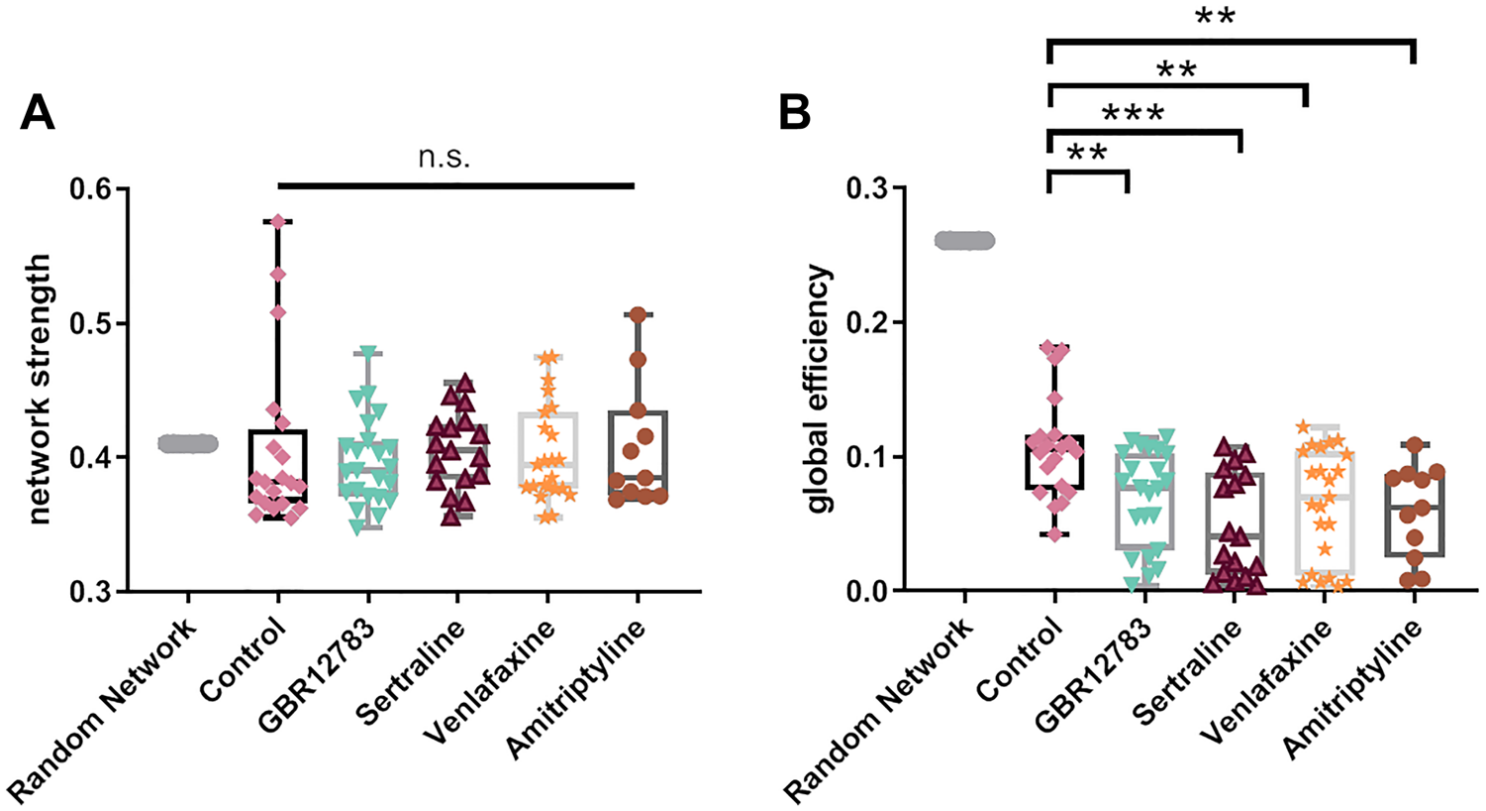
Global efficiency is reduced after monoamine reuptake inhibition. **a** Inhibition of monoamine reuptake does not lead to a change of the network strength (average connection strength in the network): GBR12783, Sertraline, Venlafaxine, Amitriptyline, and control (*n* = 20). **b** Global efficiency is significantly decreased after treatment with all tested monoamine reuptake inhibitors. *p < 0.05 after one-way ANOVA followed by Dunnett’s post hoc test. Monoamine reuptake inhibitors were present during incubation for 48 h. Data from simulated random networks were added for comparison. Sample sizes: control (*n* = 20), GBR12783 (10 µM, *n* = 23); Sertraline (1 µM, *n* = 17); Venlafaxine (50 µM, *n* = 23); Amitriptyline (10 µM, *n* = 11). The boxes extend from the 25th to the 75th percentiles. The median is shown as the horizontal line. The whiskers show the range of values

Despite yielding different detailed topological formations, we found large-scale network alterations that were common to cultures treated with the majority of the tested monoamine reuptake inhibitors. The tested different monoamine reuptake inhibitors all reduced the connectivity degree of the neuronal cultures—the number of connections formed, normalized to the number of observed cells (one-way ANOVA: F(4,89) = 6.608, *p* = 0.0001). Monoamine reuptake inhibition by GBR12783 (Post hoc Dunnett’s test: *p* = 0.018), Sertraline (Post hoc Dunnett’s test: *p* = 0.002), and Venlafaxine (Post hoc Dunnett’s test: *p* = 0.0001) reduced the number of connections in all networks, indicating a common pattern of restructuration for the network (Figs. 4A, B). We also calculated the characteristic path length (CPL), either in a binary or a connection strength-weighted fashion (Figs. 5A, B). We found that treatment with monoamine reuptake inhibitors did not induce any changes of the binary (one-way ANOVA: F(4,89) = 1.347, *p* = 0.259) and weighted characteristic path length (one-way ANOVA: F(4,89) = 1.518, *p* = 0.2036). This suggests that the lost connections stem from rather repetitive or ‘surplus’ pathways, rather than the main information transfer routes throughout the network. The network’s centrality property describes its reliance on connections that link local neighborhoods and play a ‘central’ role in the distribution of information throughout the network. Analyzing this, we found all cultures elevated centrality levels, though this change did not reach significance (Fig. 3D) (one-way ANOVA: F(4, 89) = 1.567, *p* = 0.1900). The structure of a network can be described by the modularity degree—the degree to which a network can be divided into modules (module affiliation according to a generalized Louvain community optimization (Leicht and Newman 2008)). Modules are defined as groups of cells that maximize the intra-module connectivity and minimalize the inter-module connectivity. The modularity degree did not vary much under control conditions (0.36 ± 0.07; *n* = 20; Fig. 5C), but treatment with all the tested monoamine reuptake inhibitors significantly increased network modularity, which seems counter-intuitive (one-way ANOVA: F(4,89) = 4.728, *p* = 0.0017; Dunnett’s post hoc test *p* < 0.05 for every inhibitor). The number of found modules was increased in the treatment condition, as well (see Supplementary Figure S4). An analysis of the module size, however, revealed that the networks converged towards a large number of small ‘modules’ of only two cells in the treatment conditions (see Supplementary Figure S5). As a results of this, we see an accumulation of values of 1 in the average in and out degree within individual modules (see Supplementary Figures S6 and S7). This indicates that the lost connections (Fig. 4) may be lost at the edges of the persevered modules, freeing cells of the original modular structure and leaving the network susceptible to reorganization. Despite these uniform changes of modularity, the overall network strength (average connection strength in the network) remained unaffected (one-way ANOVA: F(4,89) = 0.1979, *p* = 0.9389; Dunnett’s post hoc test *p* > 0.05 for every inhibitor; Fig. 6A). Also, the spike rate within the individual models remains constant and matches the network-wide average (see Supplementary Figure S8). Intriguingly, the global efficiency of information transfer, a measure of functional integration revealing how efficiently nodes communicate when transferring information in parallel (Rubinov and Sporns 2010; Bullmore and Sporns 2009), was reduced after treatment with all tested reuptake inhibitors (one-way ANOVA: F(4,89) = 6.284, *p* = 0.0002; Dunnett’s post hoc test *p* > 0.05 for every inhibitor, Fig. 6B). A change may also result from the diluted modular organization.

These results reveal a common pattern of lost network organization in cultures treated with monoamine reuptake inhibitors.

## Discussion

In the present study, we used fast calcium fluorescence data from live-cell imaging of in vitro hippocampal cultures to infer the underlying neuronal connectivity on a single-cell level. Investigating the effects of monoamine reuptake inhibition on the neuronal networks revealed that although different compounds may target single cells and act on neuronal networks through different pathways, we can identify a common mechanism of action—a reduction of connections that undercut the modular network structure.

Due to a lack of a reliable in vitro model of major depressive disorder, our experiments were conducted with healthy neurons on which antidepressant drugs might have little or deviant effects (Castrén and Hen 2013). However, these results give us a first insight into the underlying micro-scale neuronal network structure that forms meso- and macro-scale networks and behavioral circuits. A clear understanding of the effects of pharmacological treatment on this level will help us to understand higher level functions and to develop more specifically targeted drugs.

Another point of debate in all in vitro studies is the appropriate culturing age. Dissociated neuronal cultures exhibit electrical activity already after a few days in culture. The first isolated spiking events develop more and more towards synchronized spiking as the neuronal cultures reconnect and form networks. This goes so far that after 2–3 weeks, these cultures are driven almost exclusively by unnaturally strong and culture-wide bursting events. While some studies suggest using older cultures (Marom and Shahaf 2002; Downes et al. 2012; Chiappalone et al. 2006), activity patterns most similar to in vivo cortical activity are made up of a combination of synchronous bursting and cells’ individual spiking (Kamioka et al. 1996), as exhibited by the cultures at the age of 10 to 12 days as used in our experiments. It is noteworthy that our experiments were conducted with hippocampal neurons, whereas antidepressant drugs affect various interconnected brain areas in different ways. To reach a brain wide general conclusion on the effects of monoamine reuptake inhibition—if even possible—networks need to be studied and compared in many different brain areas.

The tested antidepressant drugs have been found to affect neuronal function through enhanced expression of synaptic proteins at the used concentrations: Sertraline 1 µM and Imipramine 10 µM (which is used in the same dosage as Amitriptyline in the clinical setting) enhanced the expression of synaptic proteins and dendritic outgrowth (Seo et al. 2014). Amitriptyline also increased the expression of neurotrophic factors at a concentration of 10 µM (Kajitani et al. 2012). Venlafaxine was able to enhance expression of genes related to cell metabolism, growth, and signaling at a concentration of 90 µM, although with a narrow range between effective and cytotoxic concentrations (de Leeuw et al. 2020). Clinically used antidepressant drugs only partly target the dopaminergic system. Since dopaminergic dysregulation is leading to some of the core symptoms of major depressive disorder (Belujon and Grace 2017), we also investigated the selective dopamine inhibitor GBR12783 in this study, although it is not used as an antidepressant drug in the clinical setting.

Patients with major depressive disorder often take the tested medications for weeks until the effects on a behavioral scale are measurable. Neuronal dynamics here were recorded after 48 h of treatment. In our previous study (Wrosch et al. 2017), we were able to show that changes on the level of neuronal networks can be detected already after this short time period. Also with this study, we were able to detect changes in network dynamics after 48 h treatment with antidepressant drugs. To fully uncover the mechanisms, however, studies with longer incubation periods will be necessary in the future. FMRI studies suggest that it takes a couple of weeks for changes to occur on a whole-brain scale (Wang et al. 2015). Nevertheless, changes on network dynamics at a micro-scale level can be detected earlier.

We found differing effects of different compounds on in-detail neuronal network topology: Only serotonin reuptake inhibition promoted strong local clustering and high vulnerability. However, we found that all tested monoamine reuptake inhibitors converged on a common pattern of action on a larger scale of network dynamics (Table 1).

**Table 1:**
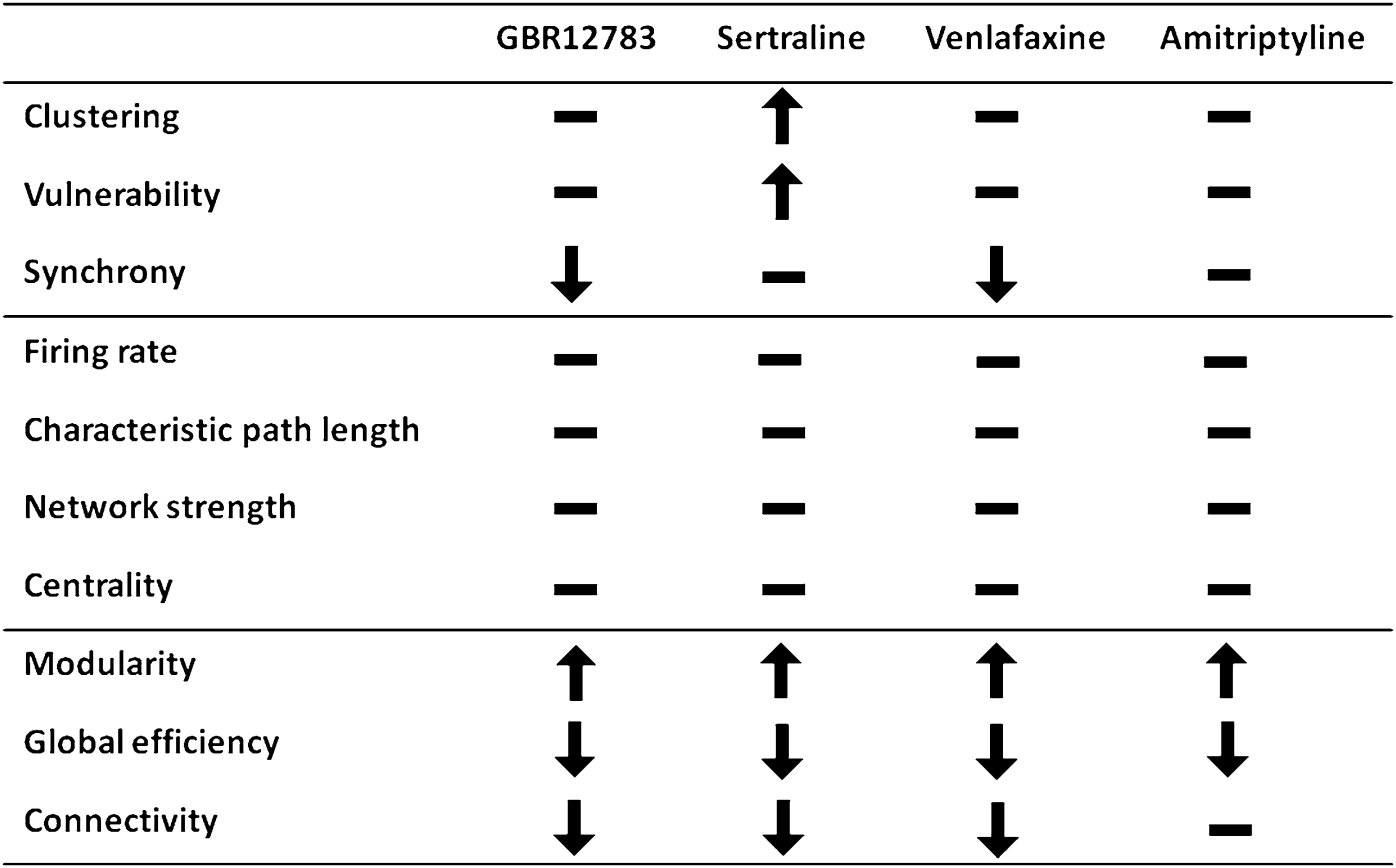
Summary of the changes in network parameters after monoamine reuptake inhibition

Cultures treated with Sertraline, Venlafaxine, Amitriptyline, and GBR12783 showed higher modularity with an accumulation of small modules, lower connectivity, and lower global efficiency. This effect is diluting the network organization in treated networks and may form the basis of a vulnerable network state that is susceptible to rewiring and ultimately behavioral change. Especially considering the short-treatment duration, what we see here, might be a transitional network state on the way to a reorganized structure.

Similar effects regarding lower connectivity could also be found in fMRI studies on a whole-brain scale where patients depicted lower functional connectivity in the hippocampus after treatment with SSRIs or SNRIs (Andreescu et al. 2013; Posner et al. 2013).

An optimally balanced and economic network consists of many specialized groups which also have sufficient connections in between to obtain high functional integration and segregation; it is also called small-world network (Latora and Marchiori 2001; Bullmore and Sporns 2009). Recent studies found that such modular small-world network structure is disturbed in psychiatric and neurological diseases. Patients suffering from major depressive disorder showed higher global efficiency and a shift towards unstructured, random network connections as compared to healthy control groups (Zhang et al. 2011; Guo et al. 2014; Hou et al. 2016; Leistedt et al. 2009). Though there are also contradictory findings (Park et al. 2014; Ye et al. 2016), other diseases like schizophrenia (Lynall et al. 2010; Rubinov et al. 2009) and Alzheimer’s Disease (Supekar et al. 2008; Stam et al. 2009) also tend towards a more random network structure than in health.

In our study, monoamine reuptake inhibitors shifted the structure towards a more modular structured network, suggesting an ability to reverse the pathological shift towards a more random network organization. The detailed mechanism of these synaptic changes is still a focus of research. A key role is most likely the enhancement of synaptic proteins. It was shown that especially BDNF is enhanced by antidepressant drugs in in vivo and in vitro experiments (Brunoni et al. 2008; Seo et al. 2014). Other key players are also PSD-95 and SYP. Seo et al. showed that different classes of antidepressant drugs elevated the expression of these synaptic proteins via a calcium/calmodulin kinase II, protein kinase A or phosphatidylinositol 3-kinase signaling way. These proteins lead to increased dendritic outgrowth and synapse formation (Seo et al. 2014). It is probable that changes of effective connectivity are mediated through modulation of a variety of synaptic proteins. However, a detailed understanding of these molecular mechanisms has yet to be achieved.

The ‘small world’ organization was proposed as the structural basis for new activity patterns and neuronal plasticity (Kaiser et al. 2007). Lower global efficiency through less connections between modules—as we found in monoamine reuptake inhibitor treated cultures—might prevent pathological activity from spreading across the whole network (Kaiser et al. 2007). Treated networks hence might be more dynamic and more susceptible to changes as it might be easier to break pathological activity patterns.

Highly connected modules within brain networks are able to promote the generation and stabilization of neuronal activity and increase the complexity of activation patterns (Okujeni et al. 2017; Fuchs et al. 2007; Kaiser et al. 2007). Sharpened networks with an increased functional variability and adaptability could be the basis for the recovery of a miswired network (Castrén and Hen 2013). Relevant connections later are stabilized, whereas weak connections are eliminated (Castrén and Hen 2013). As this selection is an active process and based on internal and external stimuli, antidepressant drugs might need to be combined with other forms of rehabilitation such as psychotherapy to reach their full potential (Castrén and Hen 2013; Colman et al. 1997). Rewiring takes time and might explain the delayed clinical effects of antidepressant drugs and the inefficiency of drug intake if further therapy strategies are lacking.

We could show here that despite some compound specific effects, different monoamine reuptake inhibitors converged on a common pathway of action. Antidepressant treatment of hippocampal cultures resulted in a reduced number of connections, increased modularity, and lower efficiency. These changes provide networks that are more susceptible for further restructuring through neuronal plasticity, medication, or psychotherapy. With this, we showed the fruitfulness of our novel approach in investigation connectivity on a single-cell basis. This method delivers a valuable puzzle piece towards a deeper understanding of the underlying mechanisms of antidepressant drugs and major depressive disorder itself. More research in this direction will help to develop more specifically targeted drugs, which are desperately needed.

## Supplementary Information

Below is the link to the electronic supplementary material.

Supplementary file1 (DOCX 927 KB)
