# Correction to: Common network effect‑patterns after monoamine reuptake inhibition in dissociated hippocampus cultures

**DOI:** 10.1007/s00702-022-02545-x

**Published:** 2022-09-28

**Authors:** Julia Trepl, Marc Dahlmanns, Johannes Kornhuber, Teja Wolfgang Groemer, Jana Katharina Dahlmanns

**Affiliations:** 1 Department of Psychiatry and Psychotherapy, Friedrich-Alexander University of Erlangen-Nürnberg, Schwabachanlage 6, 91054 Erlangen, Germany; 2 Institute for Physiology and Pathophysiology, Friedrich-Alexander University Erlangen-Nürnberg, 91054 Erlangen, Germany

## Correction to: Journal of Neural Transmission (2022) 129:261–275 10.1007/s00702-022-02477-6

The Acknowledgements section was missing from this article and should have read:

The present work was performed in (partial) fulfillment of the requirements for obtaining the degree "Dr. med."/"Dr. med. dent.

The original article has been corrected.

