# Supplementary material for: Common network effect-patterns after monoamine reuptake inhibition in dissociated hippocampus cultures
Source: J Neural Transm (Vienna). 2022 Feb 24;129(3):261–75. doi: 10.1007/s00702-022-02477-6 (PMC8930948; doi:10.1007/s00702-022-02477-6)
Supplement: Supplementary file 1 — Supplementary file1 (DOCX 927 KB) [file 702_2022_2477_MOESM1_ESM.docx]

**Supplementary Data**

**
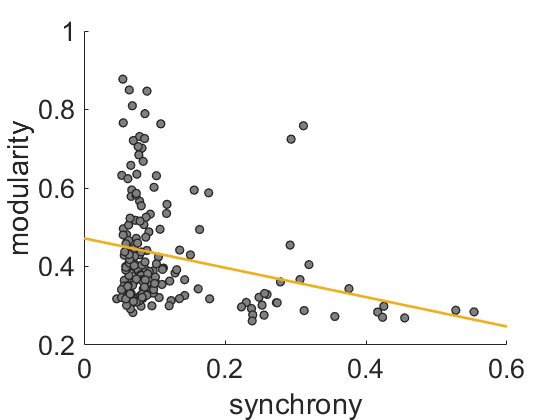
**

**Supplementary Figure S1. Neuronal activity synchrony is not correlated with reconstructed network modularity across all conditions**

Shown are the data pooled from all modules of all recordings (n=184). R² of the linear regression fit equals 0.07.

**
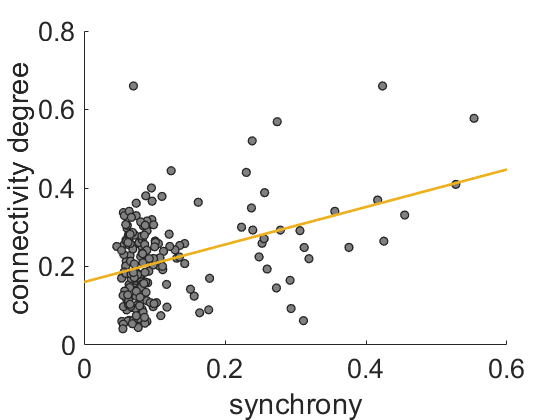
**

**Supplementary Figure S2. Weak correlation between neuronal activity synchrony and reconstructed network connectivity degree across all conditions**

Shown are the data pooled from all modules of all recordings (n=184). R² of the linear regression fit equals 0.2.

**
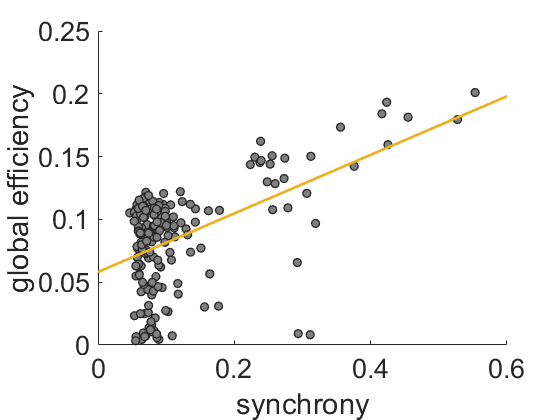
**

**Supplementary Figure S3. Weak correlation between neuronal activity synchrony and reconstructed network global efficiency across all conditions**

Shown are the data pooled from all modules of all recordings (n=184). R² of the linear regression fit equals 0.3.

**Supplementary Figure S4. Number of modules with weighted Louvain community algorithm**

Cultures treated with monoamine reuptake inhibitors showed a higher number of modules. The effect reached statistical significance for Sertraline and Venlafaxine treatment. * p< 0.05 after one-way ANOVA followed by Dunnett’s post-hoc test. Monoamine reuptake inhibitors were present during incubation for 48h. Sample sizes: control (n=20), GBR12783 (10 µM, n=23); Sertraline (1 µM, n=17); Venlafaxine (50µM, n=23); Amitriptyline (10 µM, n=11). The boxes extend from the 25^th^ to the 75^th^ percentiles. The median is shown as the horizontal line. The whiskers show the range of values.

**Supplementary Figure S5. Number of cells within modules**

Treated cultured were comprised of significantly smaller modules – many consisting of only two cells. * p< 0.05 *** p<0.001 after one-way ANOVA followed by Dunnett’s post-hoc test. Monoamine reuptake inhibitors were present during incubation for 48h. Sample sizes: control (n=20), GBR12783 (10 µM, n=23); Sertraline (1 µM, n=17); Venlafaxine (50µM, n=23); Amitriptyline (10 µM, n=11). The boxes extend from the 25^th^ to the 75^th^ percentiles. The median is shown as the horizontal line. The whiskers show the range of values.

**Supplementary Figure S6. In degree within modules after treatment with antidepressant drugs**

The in-degree describes the number of incoming connections of a given node. Modules were detected and for each module a mean in-degree of all nodes within the modules was calculated. All treated cultures showed a decrease of in-degree within formed modules due to an accumulation of values of one in modules of two cells. *** p<0.001 after one-way ANOVA followed by Dunnett’s post-hoc test. Monoamine reuptake inhibitors were present during incubation for 48h Sample sizes: control (n=20), GBR12783 (10 µM, n=23); Sertraline (1 µM, n=17); Venlafaxine (50µM, n=23); Amitriptyline (10 µM, n=11). The boxes extend from the 25^th^ to the 75^th^ percentiles. The median is shown as the horizontal line. The whiskers show the range of values.

**Supplementary Figure S7. Out degree within modules after treatment with antidepressant drugs**

The out-degree describes the number of outgoing connections of a given node. Modules were detected and for each module the mean out-degree for each node within the module was calculated. All treated cultures showed a decrease of in-degree within formed modules due to an accumulation of values of one in modules of two cells. *** p<0.001 after one-way ANOVA followed by Dunnett’s post-hoc test. Monoamine reuptake inhibitors were present during incubation for 48h Sample sizes: control (n=20), GBR12783 (10 µM, n=23); Sertraline (1 µM, n=17); Venlafaxine (50µM, n=23); Amitriptyline (10 µM, n=11). The boxes extend from the 25^th^ to the 75^th^ percentiles. The median is shown as the horizontal line. The whiskers show the range of values.

**Supplementary Figure S8. Spike rate within modules**

Spike rates within modules were significantly lower in cultures treated with Amitriptyline and significantly higher in cultures treated with venlafaxine. However there was only a slight difference between those groups. ** p<0.01, *** p<0.001 after one-way ANOVA followed by Dunnett’s post-hoc test. Monoamine reuptake inhibitors were present during incubation for 48h Sample sizes: control (n=20), GBR12783 (10 µM, n=23); Sertraline (1 µM, n=17); Venlafaxine (50µM, n=23); Amitriptyline (10 µM, n=11). The boxes extend from the 25^th^ to the 75^th^ percentiles. The median is shown as the horizontal line. The whiskers show the range of values.
